# Supplementary material for: A handheld photoacoustic microscopic probe integrating a transparent ultrasound transducer and a fiber scanner
Source: Nat Commun. 2025 Dec 31;17:1409. doi: 10.1038/s41467-025-68148-8 (PMC12881618; doi:10.1038/s41467-025-68148-8)
Supplement: Supplementary file 1 — Supplementary Information [file 41467_2025_68148_MOESM1_ESM.pdf]

Supplementary Information for "A handheld photoacoustic microscopic probe integrating a transparent ultrasound transducer and a fiber scanner"

Mingyu Ha[1,2†], Jaewoo Kim[1,2†], Jihye Lee[3], Seonghee Cho[2,4], Dasom Heo[1,2], Minsu Kim[1,2], Joongho Ahn[2,4,6], Eunwoo Park[1,2], Joo Young Kweon[5], Yuri Kang[1], Yong Joo Ahn[1,2,5], Hyung Ham Kim[1,2,4,5], Won Jong Kim[3] and Chulhong Kim[1,2,4,5,6]*

[1]Department of Convergence IT Engineering, Pohang University of Science and Technology, Pohang, Republic of Korea
[2]Medical Device Innovation Center, Pohang University of Science and Technology, Pohang, Republic of Korea
[3]Department of Chemistry, Pohang University of Science and Technology, Pohang, Republic of Korea
[4]Department of Electrical Engineering, Pohang University of Science and Technology, Pohang, Republic of Korea
[5]Department of Medical Science and Engineering, School of Convergence Science and Technology, Pohang University of Science and Technology, Pohang, Republic of Korea
[6]Opticho Co., Ltd, Pohang, Republic of Korea

[†]These authors have contributed equally: Mingyu Ha, Jaewoo Kim.
[*]Corresponding author.
E-mail: chulhong@postech.edu

Contents

Supplementary Table. S1. Comprehensive comparison with other handheld photoacoustic probes

Supplementary Fig. S1. Simulated properties of TUTs made of LNO (blue), PMN-PT (red,) and PVDF (green)

Supplementary Fig. S2. Simulated and measured acoustic intensity field

Supplementary Fig. S3. Fill factor variation with increasing scanning frequency.

Supplementary Fig. S4. Scanning trajectory measured using various scanning frequencies.

Supplementary Fig. S5. Imaging time versus fill factor graph using fixed PRF.

Supplementary Fig. S6. Comparative imaging results between fixed PRF mode and variable PRF mode.

Supplementary Fig. S7. DAQ timing diagram

Supplementary Fig. S8. Position sensor based image reconstruction

Supplementary Fig. S9. Lateral resolution and SNR curves measured in water and tissue-mimicking phantoms.

Supplementary Fig. S10. Signal intensity map across the FOV.

Supplementary Fig. S11. Scanning trajectory measured in position sensor using 333 Hz.

Supplementary Fig. S12. Masks and skeletons for quantitative analysis of epinephrine-induced vasoconstriction

Supplementary Fig. S13. Masks and skeletons for quantitative analysis of mouse ear lymphatic vessel visualization

Supplementary Fig. S14. Masks and skeletons for quantitative analysis of vasculatures in metastatic tumors in a mouse's abdomen

Supplementary Fig. S15. Schematic of the TUT fabrication process

Supplementary Fig. S16. Photoacoustic signal comparison with and without Teflon film.

Supplementary Fig. S17. Grid target image

Supplementary Text 1. Comparative simulation of the performances of LNO-, PMN-PT-, and PVDF-based TUTs

Supplementary Text 2. Laser safety in in vivo test

Supplementary Text 3. Imaging performance test in water and tissue-mimicking phantoms.

Supplementary Text 4. Quantitative analysis of epinephrine-induced vasoconstriction in a mouse ear

Supplementary Text 5. Segmentation and quantitative analysis of mouse ear lymphatic vasculature

Supplementary Text 6. Quantitative analysis of tumor vascular architecture

	Scanner	Optical scanning	Size	FOV	Imaging speed	Resolution [µm]	Application
**K. Park et al. (2017)**[46]	MEMS	X	Ø 17 mm x 120 mm	2 x 2 mm^2	20s / volume	L : 12 A : 30	Human mole
**L. Lin et al. (2017)**[47]	MEMS	O	80 x 115 x 150 mm^3	2.5 x 2.0 mm^2	0.5s / volume	L : 5 A : 26	Mouse ear, Human mole
**Q. Chen et al. (2018)**[45]	MEMS	O	22 x 30 x 13 mm^3	2 x 2 mm^2	5s / volume	L : 3.8 A : 104	Rat internal organs, Human oral cavity
**W. Zhang et al. (2020)**[39]	MEMS	O	Ø 12 mm	Ø 2.4 mm	4s / volume	L : 18.2 A : 137.4	Human oral cavity
**W. Qin et al. (2021)**[37]	Galvanometer + rotator	O	NM	Ø 10 mm	5s / volume	L : 15 A : 120	Rhesus cerebral cortex
**J. Chen et al. (2022)**[36]	Galvanometer + MEMS	X	59 x 30 x 44 mm^3	1.7 x 5 mm^2<br>1.7 x 2 mm^2	0.5s / volume<br>0.22s / volume	L : 6.2 A : 39	Rat internal organs, mouse brain stroke, human lip
**D. Ke et al. (2023)**[35]	Fiber scanner	O	Ø 5 mm	Ø 3 mm	2s / volume	L : 15.6 A : 168	Rat stomach, intestine
**This work**	Fiber scanner	O	Ø 17 mm x 90 mm	Ø 2.6 mm	1.5s / volume	L : 7 A : 47	Rat internal organs, mouse lymphatic vessel, mouse metastatic tumor

Supplementary Table. S1. Comprehensive comparison with other handheld photoacoustic probes. FOV, field of view; L, lateral resolution; and A, axial resolution.

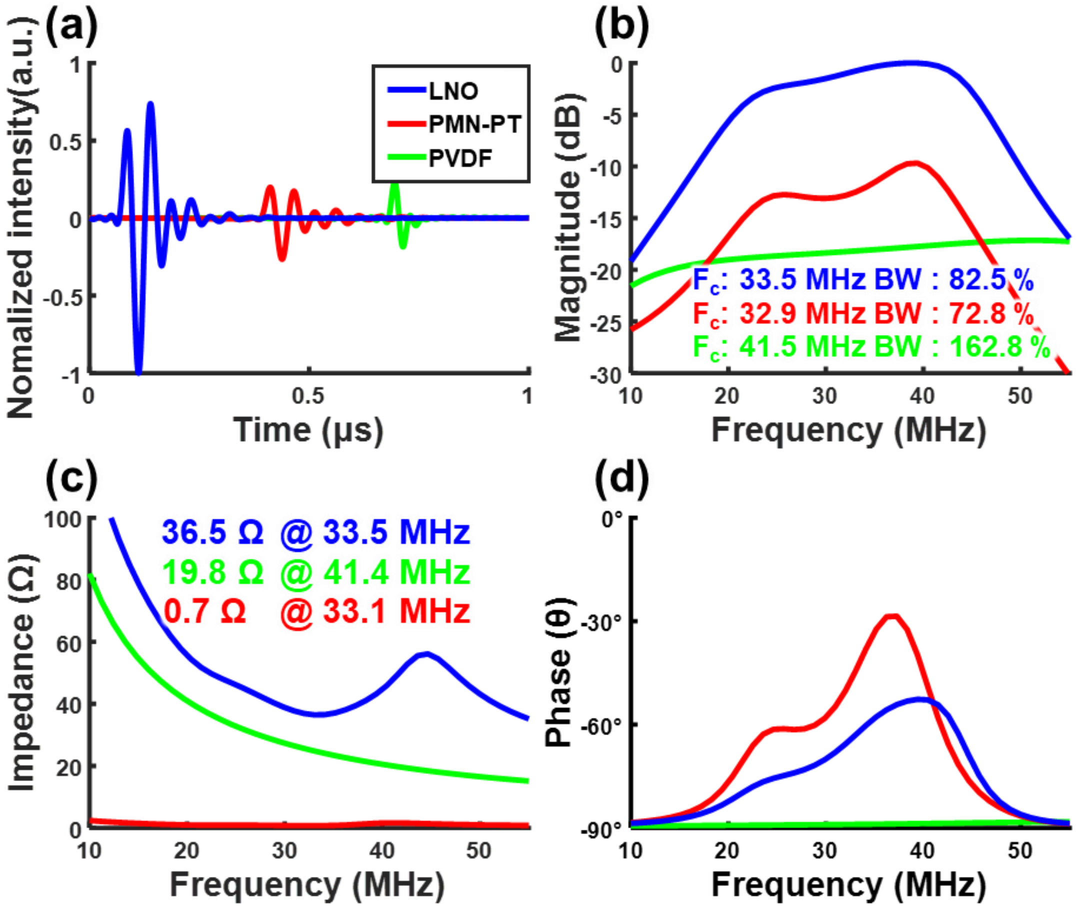

Supplementary Fig. S1. **Simulated properties of TUTs made of LNO (blue), PMN-PT (red), and PVDF (green).** One-way impulse reception of the TUTs in **(a)** the time domain and **(b)** the frequency domain. **(c)** Electrical impedance curves of the TUTs. **(d)** Phase curves of the TUTs. Fc, center frequency; BW, bandwidth.

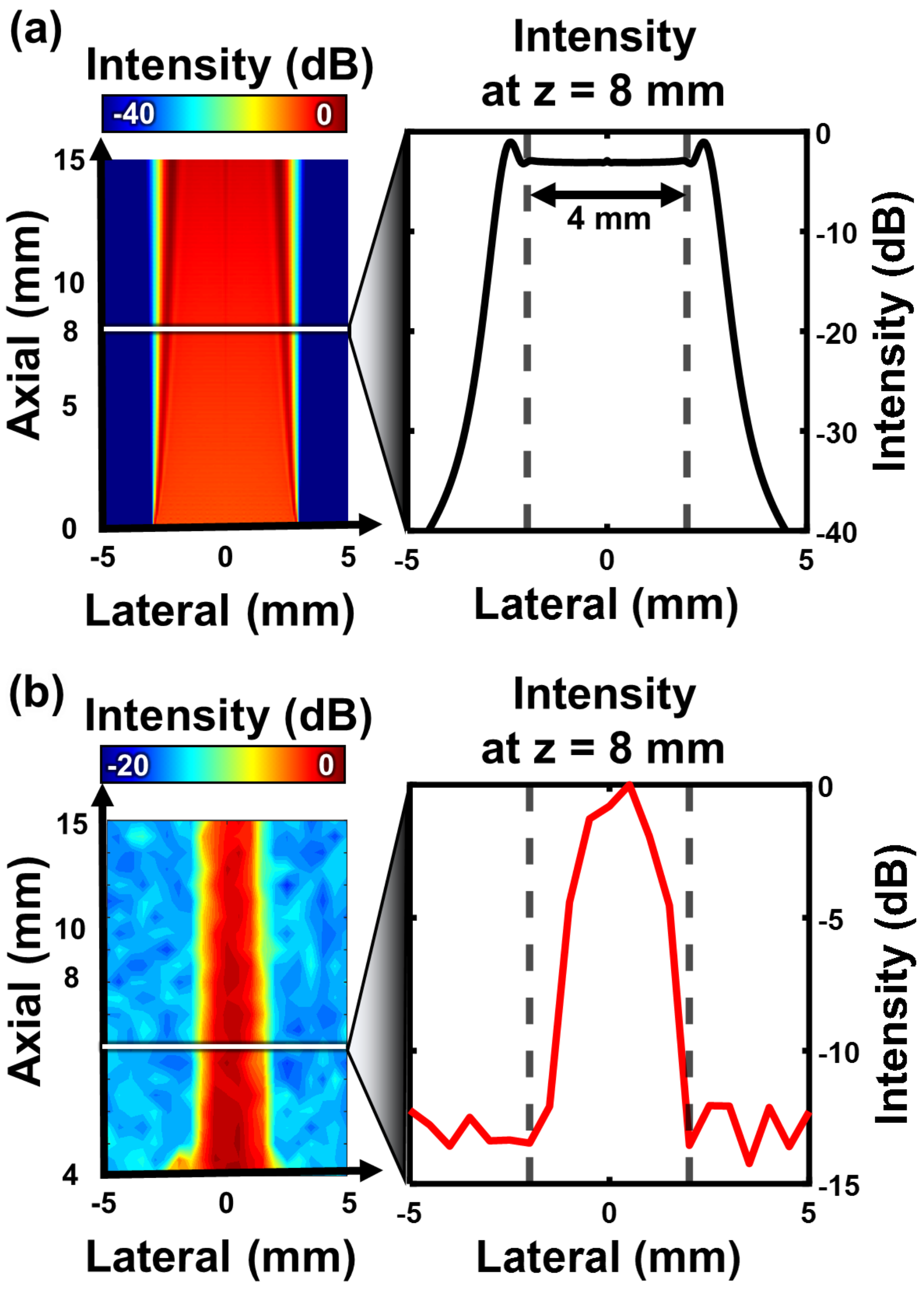

Supplementary Fig. S2. Simulated and measured acoustic intensity field. (a) Simulated acoustic field. (b) Measured acoustic field.

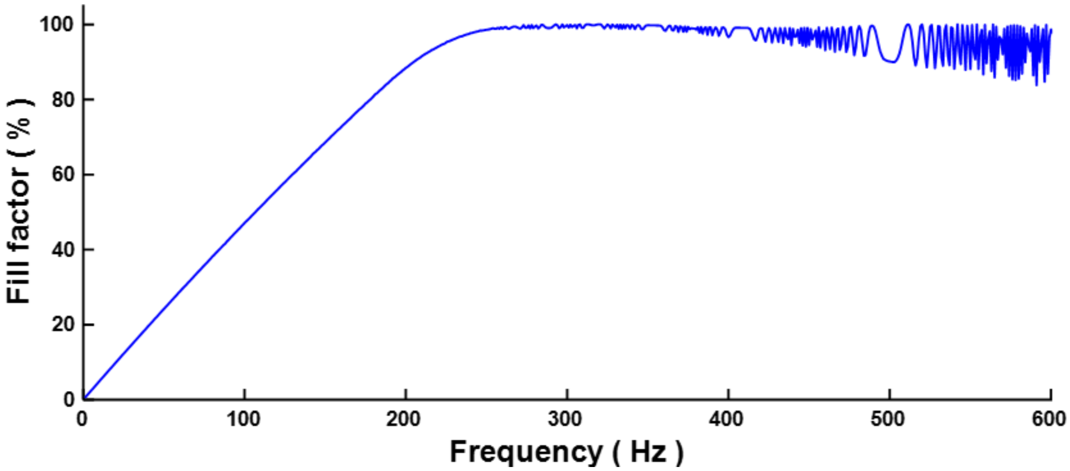

Supplementary Fig. S3. **Fill factor variation with increasing scanning frequency.** The simulation was conducted assuming the use of a 500 kHz laser and total pixel of Ø 512. Fill factor = filled pixels / total pixels × 100.

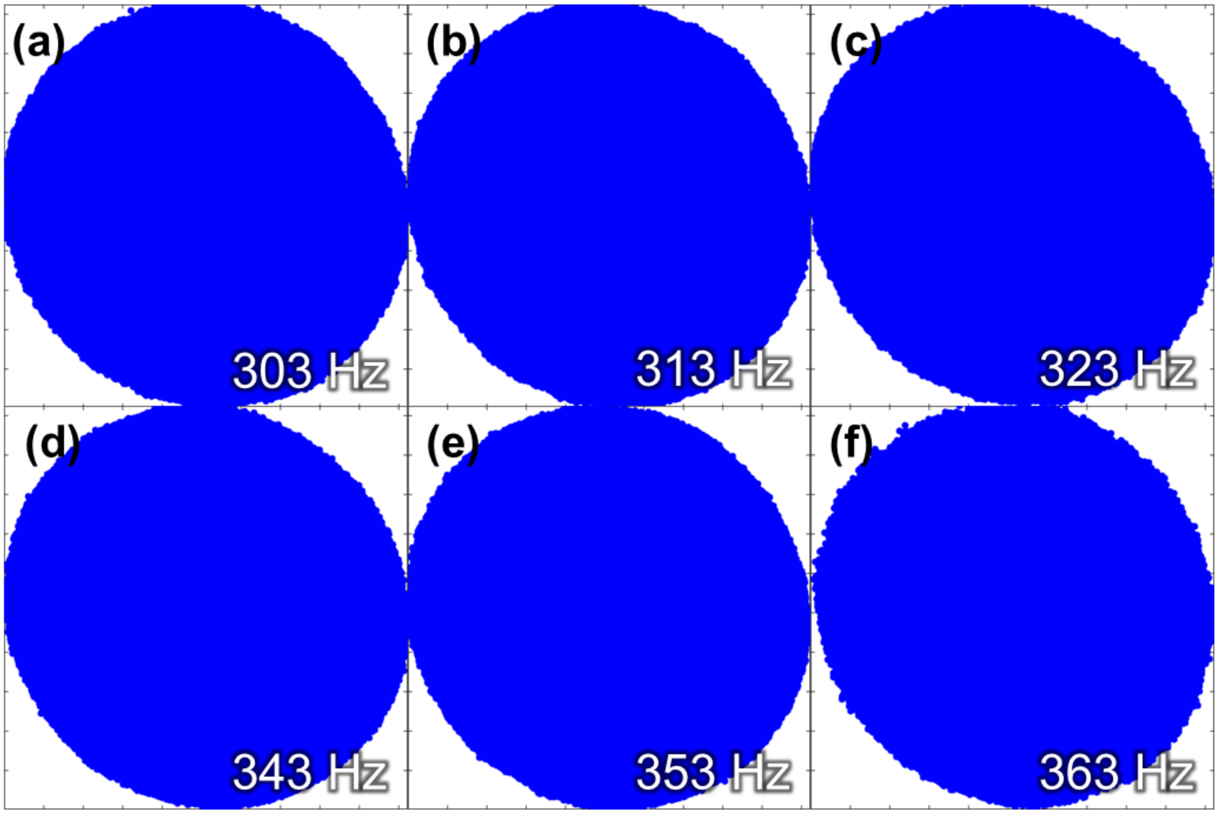

Supplementary Fig. S4. Scanning trajectory measured using various scanning frequencies. Scanning trajectory measured using (a) 303 Hz, (b) 313 Hz, (c) 323 Hz, (d) 343 Hz, (e) 353 Hz, (f) 363 Hz.

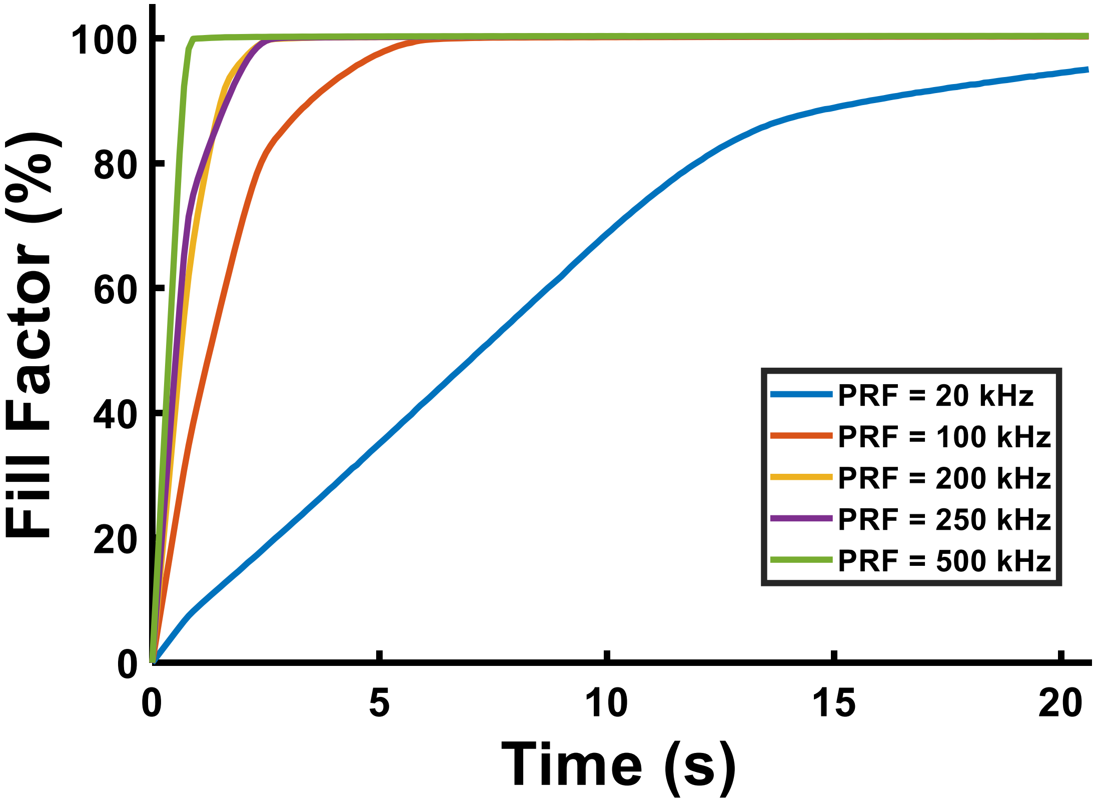

Supplementary Fig. S5. Imaging time versus fill factor graph using fixed PRF.

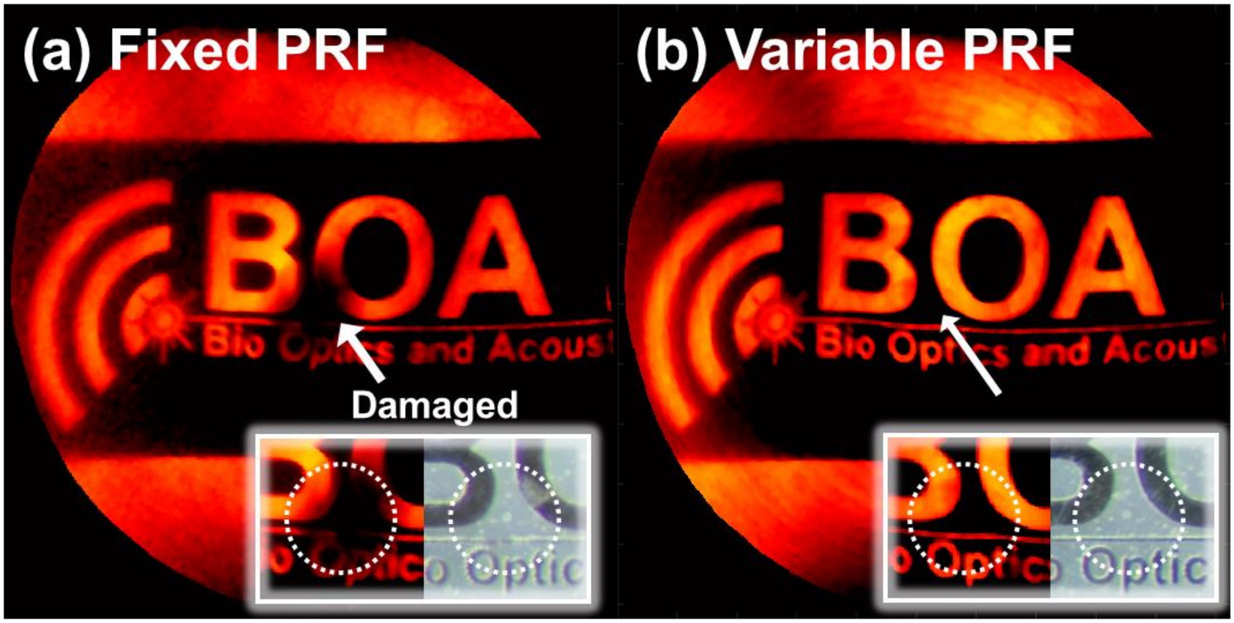

Supplementary Fig. S6. Comparative imaging results between fixed PRF mode and variable PRF mode. Image of a film target using (a) fixed PRF mode and (b) variable PRF mode.

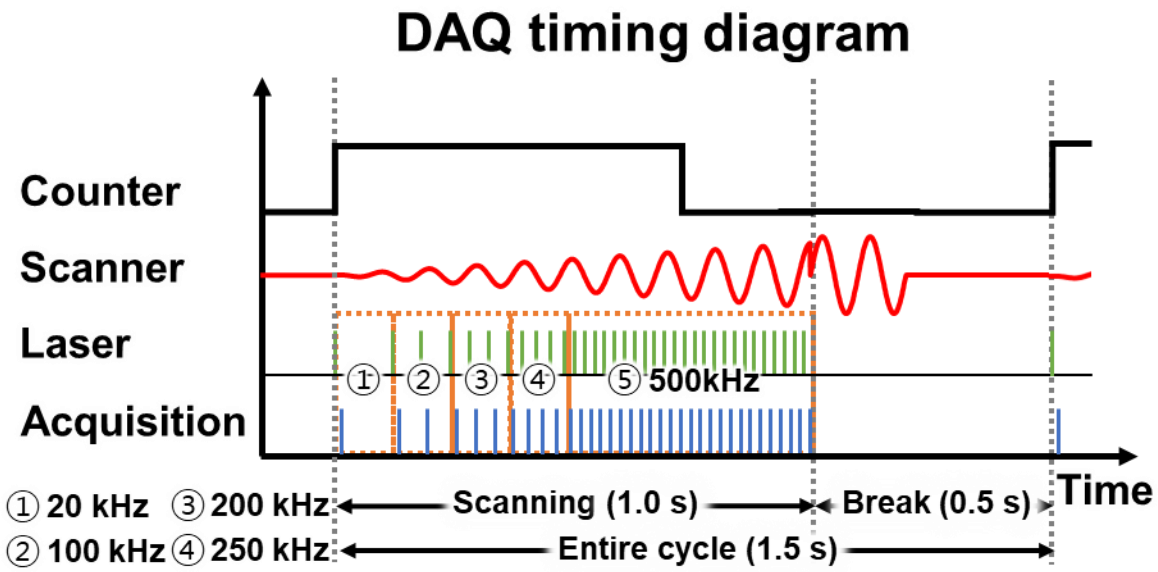

Supplementary Fig S7. **DAQ timing diagram**

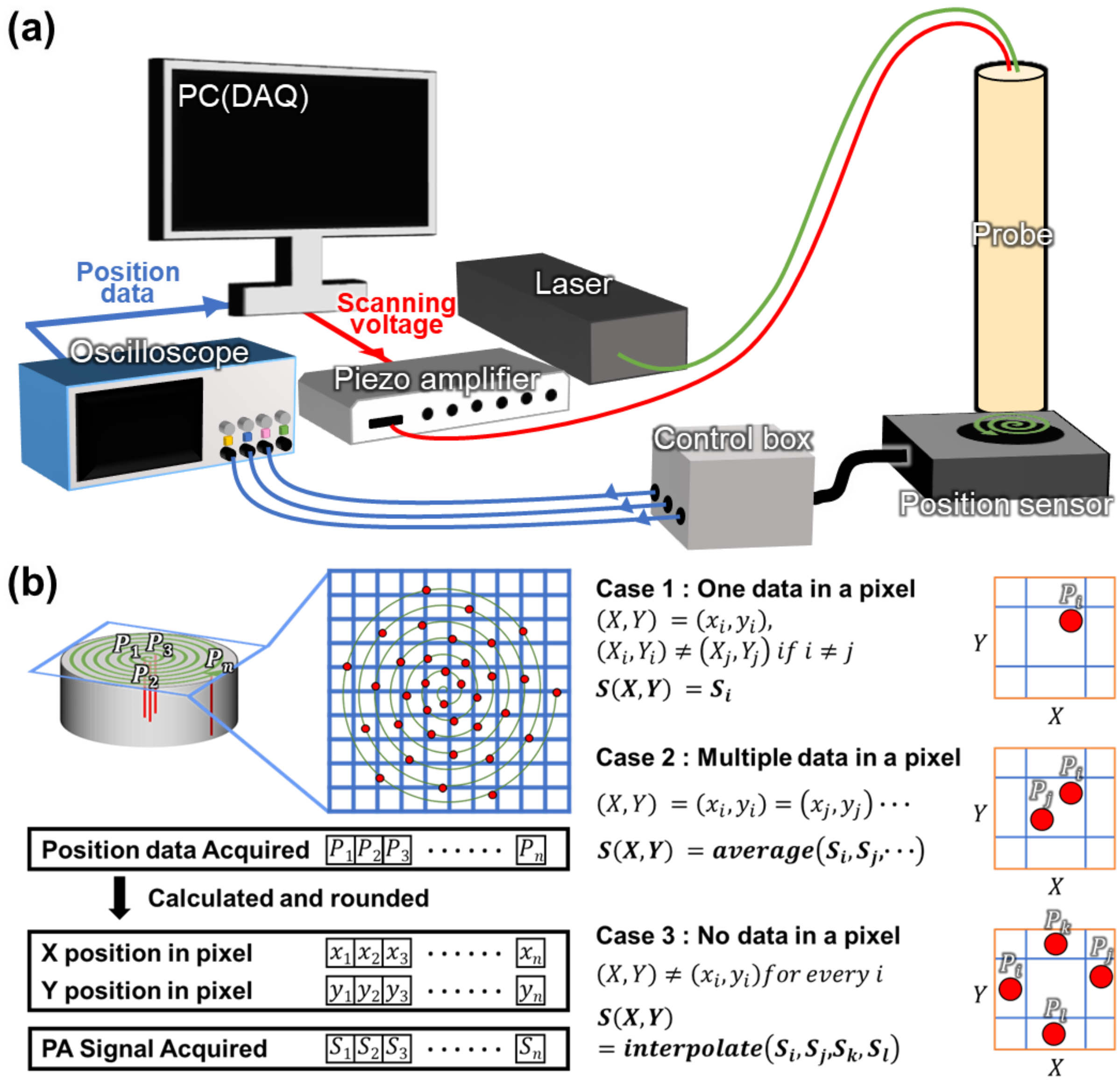

Supplementary Fig S8. **Position sensor based image reconstruction.** **(a)** Schematic of system for position sensor data acquisition. **(b)** Image reconstruction algorithm.

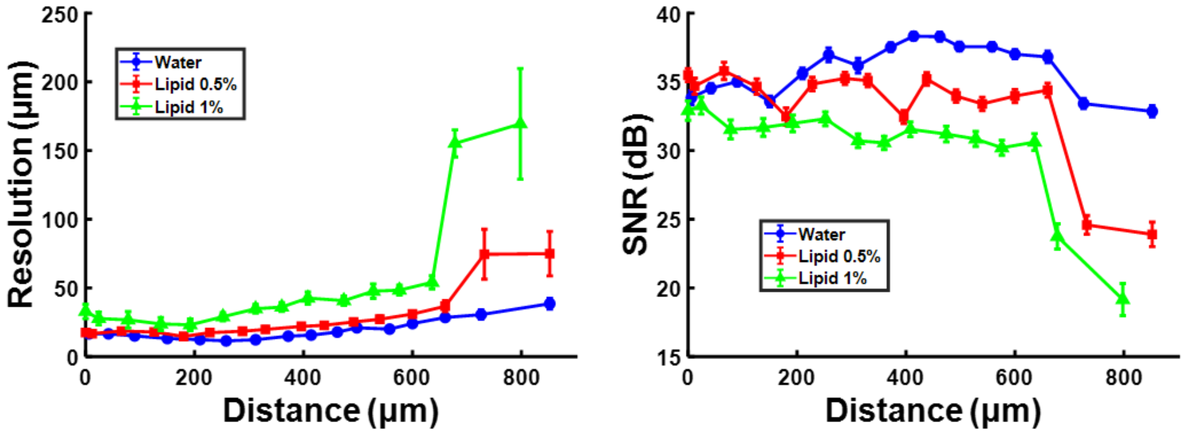

Supplementary Fig. S9. Lateral resolution and SNR curves measured in water and tissue-mimicking phantoms. (a) Lateral resolution curves, and (b) SNR curves measured in water, intralipid-0.5%, and intralipid-1.0%.

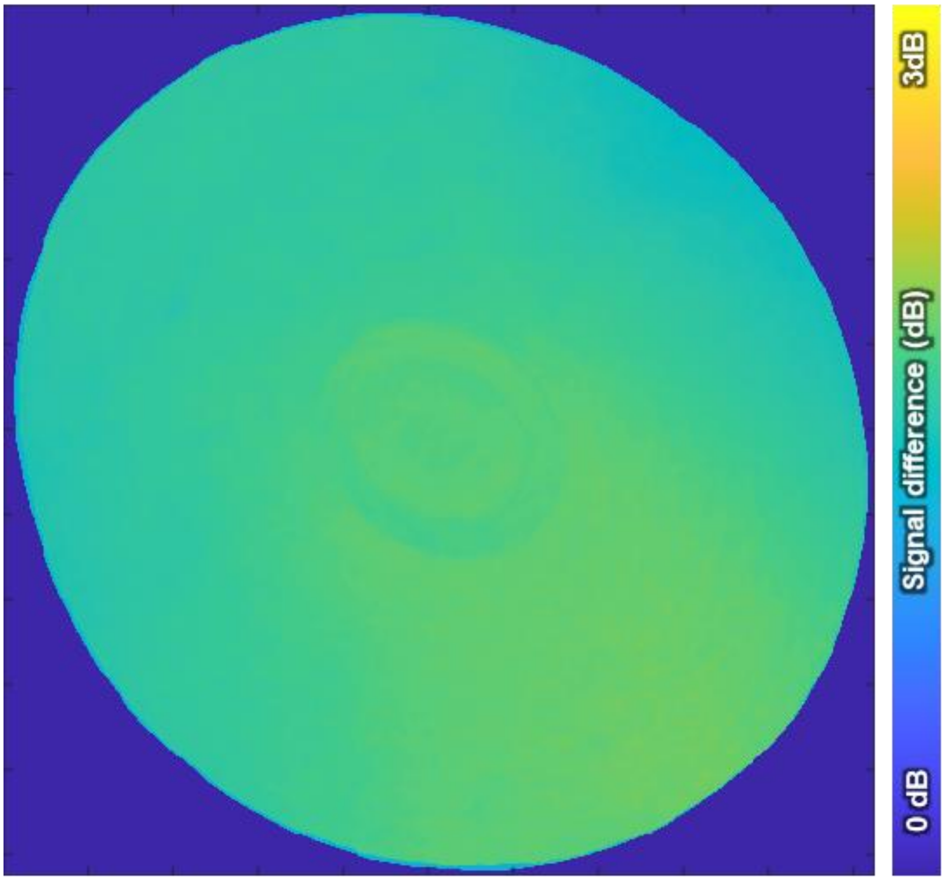

Supplementary Fig. S10. Signal intensity map across the FOV.

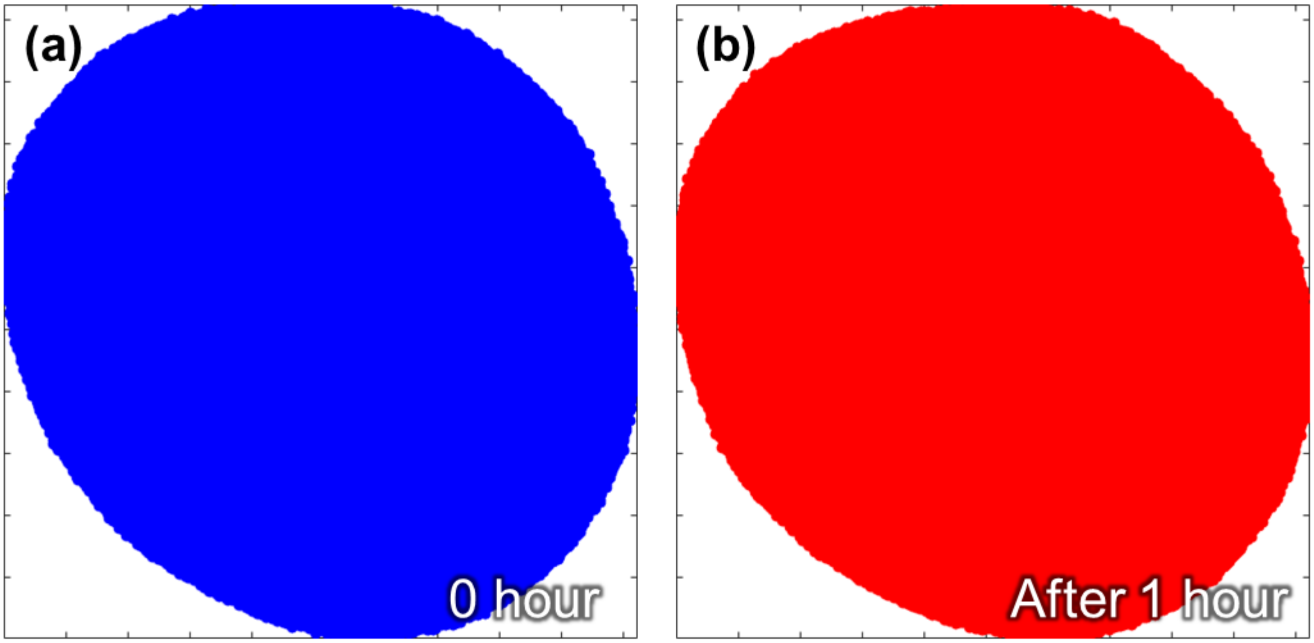

Supplementary Fig. S11. Scanning trajectory measured in position sensor using 333 Hz. Scanning trajectory (a) before and (b) after continuous 1 hour operation.

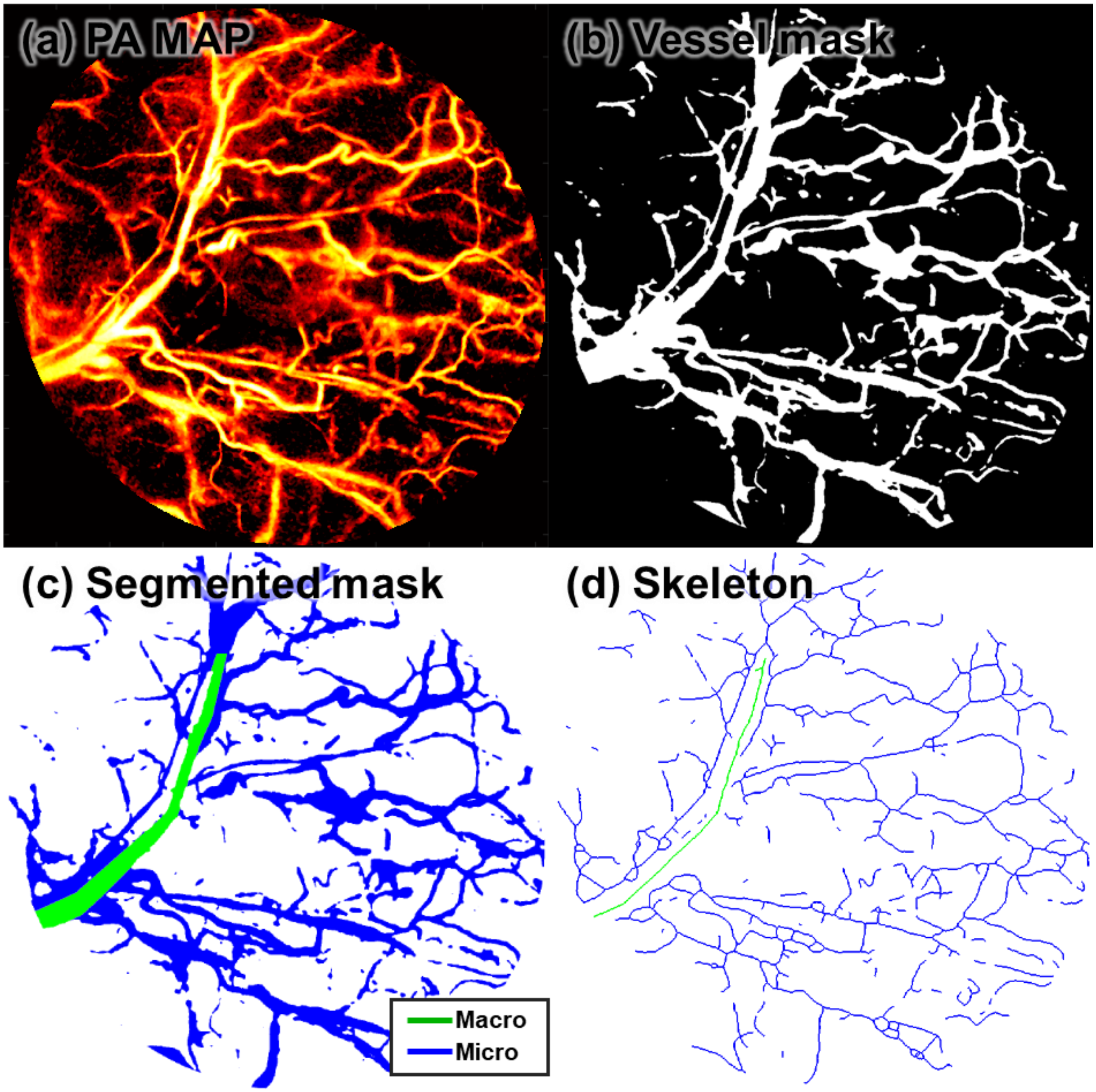

Supplementary Fig S12. **Masks and skeletons for quantitative analysis of epinephrine-induced vasoconstriction. (a)** PA MAP image. **(b)** Vessel mask. **(c)** Segmented mask. **(d)** Skeleton of segmented mask.

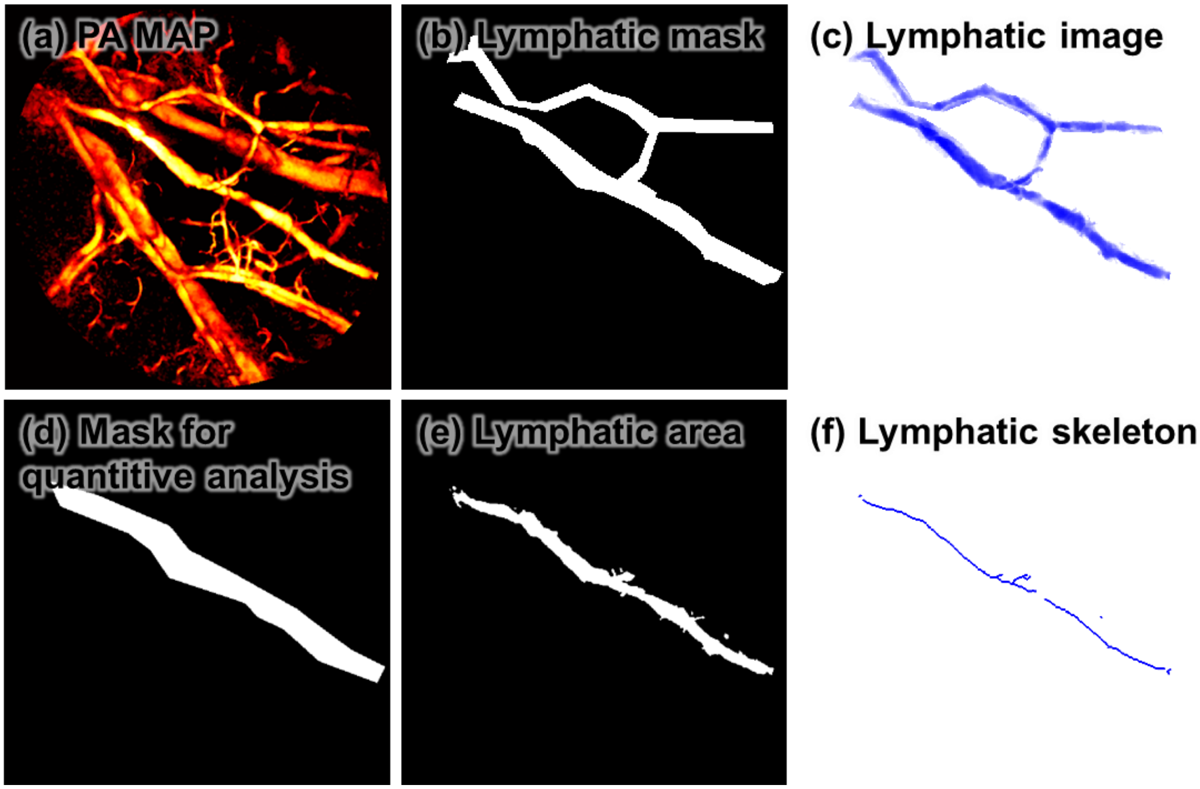

Supplementary Fig S13. **Masks and skeletons for quantitative analysis of mouse ear lymphatic vessel visualization. (a)** PA MAP image. **(b)** Lymphatic vessel mask. **(c)** Lymphatic image **(d)** Lymphatic mask for quantitative analysis. **(e)** Lymphatic area. **(f)** Lymphatic skeleton.

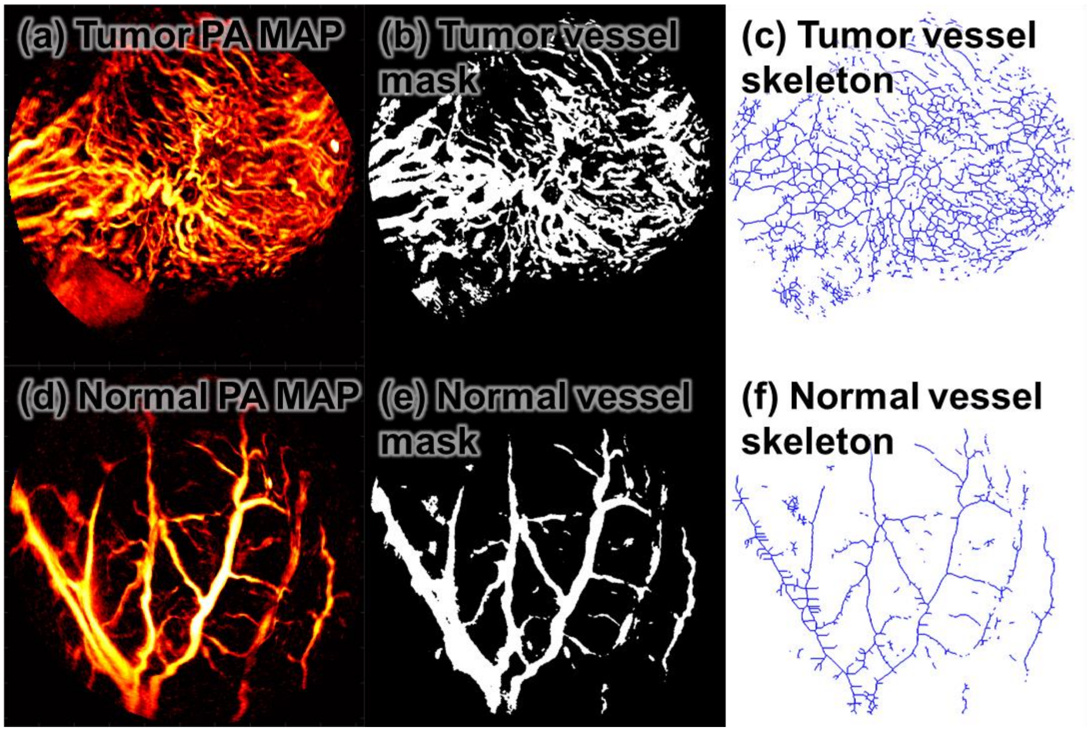

Supplementary Fig S14. **Masks and skeletons for quantitative analysis of vasculatures in metastatic tumors in a mouse's abdomen. (a)** PA MAP image of the tumor area. **(b)** Vessel mask of (a). **(c)** Vessel skeleton of (a). **(d)** PA MAP image of the normal area. **(e)** Vessel mask of (d) **(f)** Vessel skeleton of (d).

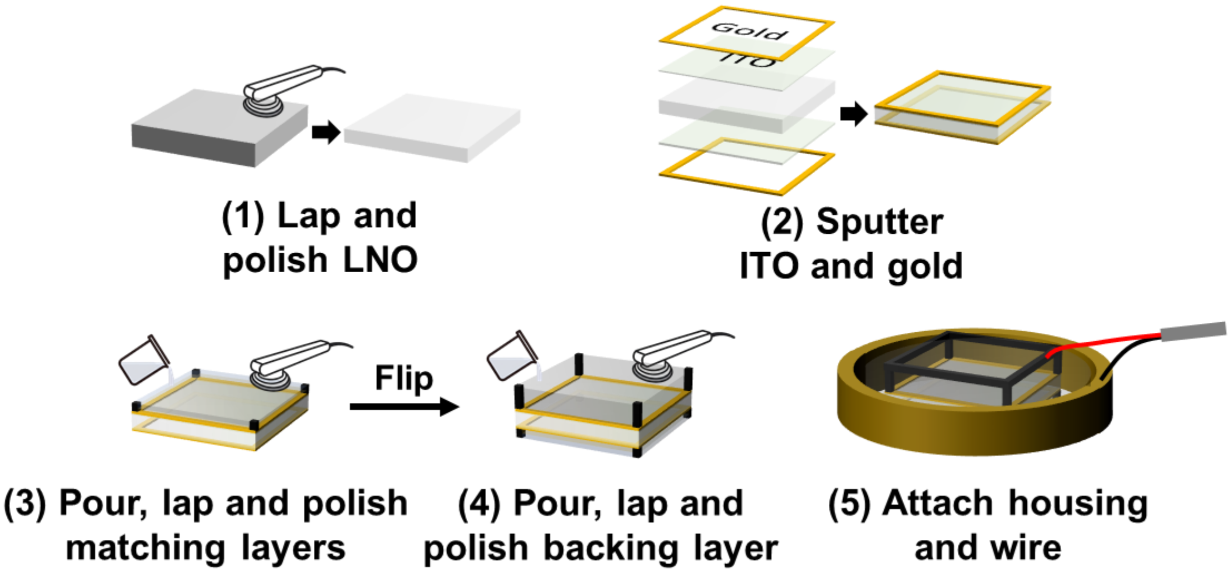

Supplementary Fig S15. **Schematic of TUT fabrication process**

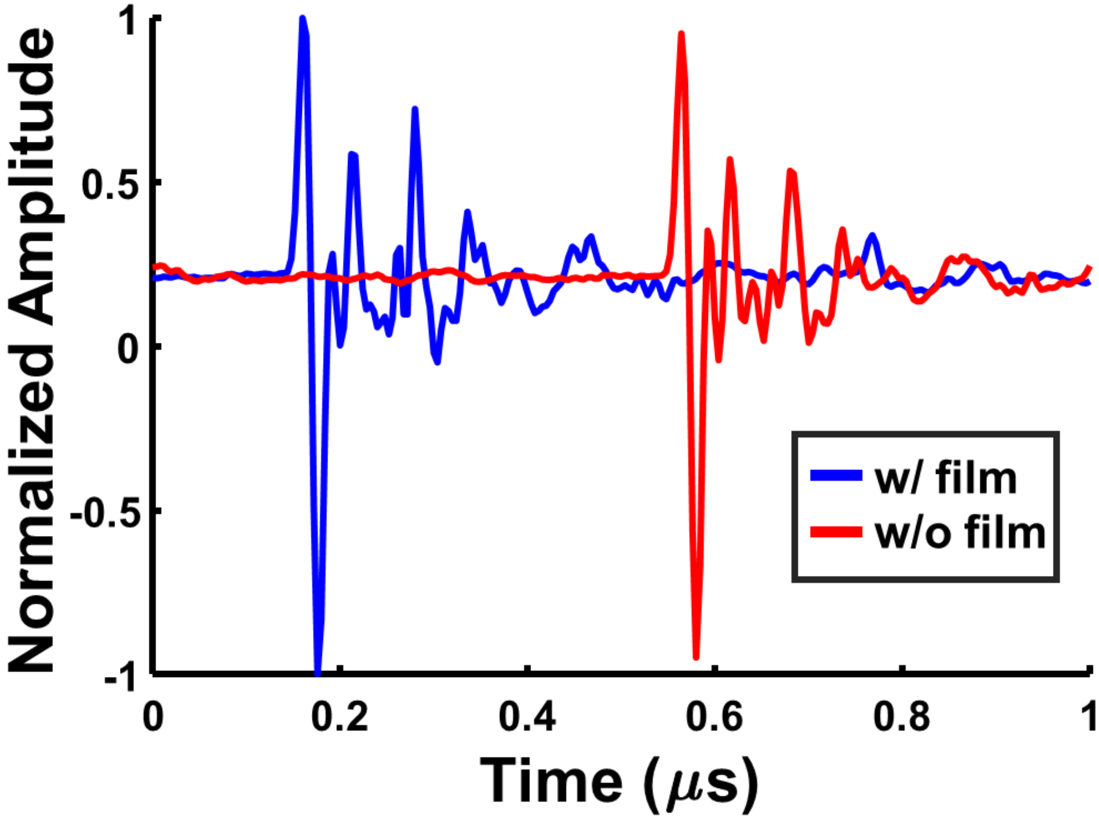

137

138 **Supplementary Fig. S16. Photoacoustic signal comparison with and without Teflon film.**

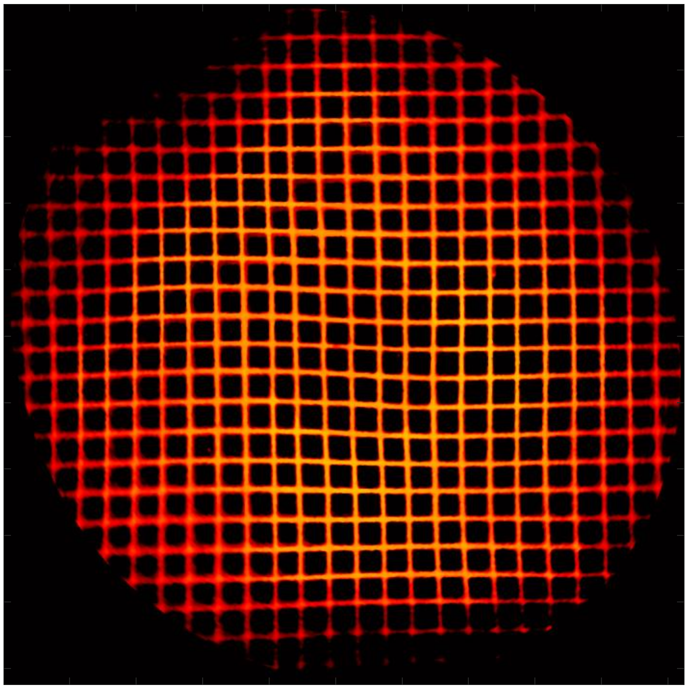

Supplementary Fig. S17. Grid target image

Supplementary Texts

Supplementary Text 1. Comparative simulation of the performances of LNO-, PMN-PT-, and PVDF-based TUTs

Although LNO, PMN-PT, and PVDF have all been used for TUT fabrication, when it comes to high-frequency transducers for handheld probes, each material exhibits distinct limitations. To illustrate this, we simulated the performances of TUTs made of PMN-PT, PVDF, and LNO with a size of 6 mm × 6 mm along the x and y axes and a center frequency near 35 MHz. Fig. S1(a) shows the simulated one-way receiving performances for these TUTs in the time domain. The LNO-based transducer produces the highest signal response, while the PMN-PT and PVDF devices achieve only about 25.9% and 23.5% of the LNO's response, respectively. In the frequency domain (Fig. S1(b)), the PVDF transducer exhibits a center frequency of 41.5 MHz and an impressive bandwidth of 162.8%, yet its signal amplitude is approximately 20 dB lower than that of the LNO device. In contrast, the PMN-PT transducer shows a center frequency of 32.9 MHz, a bandwidth of 72.8%, and a signal amplitude roughly 10 dB below LNO's. These performance differences arise from the intrinsic properties of the materials. PVDF's low piezoelectric charge coefficient (d33) leads to reduced voltage conversion efficiency under equal pressure, resulting in a smaller signal output. Meanwhile, PMN-PT's high dielectric constant causes its capacitance to increase substantially with size, thereby lowering the voltage output. Furthermore, as shown in Fig. S1(c), the PMN-PT transducer has an extremely low electrical impedance (approximately 0.7 Ω at its center frequency), making it challenging to match with standard 50 Ω systems and hindering efficient signal transfer. In summary, due to the low voltage output from the PVDF and the impedance mismatch issues associated with the PMN-PT, both materials yield inferior signal amplitudes in the simulated high-frequency transducer design with a size of 6 mm × 6 mm. Consequently, LNO emerges as the most suitable piezoelectric material for high frequency TUTs for handheld probe applications.

Supplementary Text 2. Laser safety in in vivo test

In all in vivo experiments of this study, the laser pulse energy was 600 nJ. The focal point of the system was formed about 250 μm beneath the film in the water tank; however, during actual imaging with tissue contact, the film was slightly compressed, and the focus was formed at approximately 300 μm within the tissue. Based on an NA of 0.18, the beam spot diameter at the tissue surface was estimated to be ~82 μm, corresponding to an energy density of ~11.3 mJ/cm^2. This value is below the ANSI MPE" limit for skin (20 mJ/cm^2), indicating a high level of photothermal safety for clinical applications.

Supplementary Text 3. Imaging performance test in water and tissue-mimicking phantoms

To evaluate the imaging performance in tissue, we measured the lateral resolution and signal-to-noise ratio (SNR) at various depths in both water and tissue-mimicking phantoms (Intralipid 0.5% and Intralipid 1%) (Fig. S9). A printed film served as the imaging target. The edge of the printed film was imaged, and the line spread function (LSF) was derived from the edge spread function (ESF). The full width at half maximum (FWHM) of the LSF was calculated to determine the lateral resolution. The highest lateral resolutions were 11.5 μm, 14.7 μm, and 23.2 μm in water, Intralipid 0.5%, and Intralipid 1%, respectively. SNR was measured from the same target, with maximum values of 38.2 dB, 37.6 dB, and 35.0 dB, which gradually decreased with imaging depth to 32.9 dB, 23.9 dB, and 19.2 dB in water, Intralipid 0.5%, and Intralipid 1%, respectively. These results

indicate that both resolution and SNR degrade with increasing scattering, as expected in tissue-mimicking media. Beyond ~800 µm, the resolution degrades and the SNR decreases sharply, limiting the maximum imaging depth to ~800 µm.

Supplementary Text 4. Quantitative analysis of epinephrine-induced vasoconstriction in a mouse ear

Fig. S12(a) displays a PA MAP image of a mouse's ear vasculature prior to epinephrine injection. A threshold was applied to this image to create a vessel mask (Fig. S12(b)). Subsequently, the thickest portion of each vessel was measured from the PA MAP image. Vessels with a thickness exceeding 100 µm were manually segmented as macro vessels, while the remainder were classified as micro vessels (Fig. S12(c)). Based on these masks, changes in vessel density over time were quantified. Fig. S12(d) shows the skeleton extracted from the segmented mask, and the average signal intensity along these skeleton positions was used to quantitatively monitor temporal changes in PA amplitude.

Supplementary Text 5. Segmentation and quantitative analysis of mouse ear lymphatic vasculature

Fig. S13(a) shows a PA MAP image of a mouse ear acquired 3 seconds after an Evans blue injection. The pre-injection 3D data were subtracted from the post-injection 3D data, and the resulting dataset was mapped. Based on the subtracted image, a lymph mask was manually created (Fig. S13(b)). Fig. S13(c) presents the lymph image obtained by applying the lymph mask to the PA MAP image. These images were subsequently overlaid with the PA MAP to produce the images shown in Fig. 7(a). For lymph area and lymph signal intensity analysis, a mask was manually delineated by selecting lymph vessels in regions that did not overlap with blood vessels (Fig. S13(d)). This mask was then applied to the PA MAP, and thresholding was used to define the lymph area for area change analysis (Fig. S13(e)). Further, the skeleton of the lymph area was extracted (Fig. S13(f)), and the average PA signal intensity along the skeleton was used to quantitatively assess the temporal changes in PA amplitude.

Supplementary Text 6. Quantitative analysis of tumor vascular architecture

Fig. S14(a) shows a PA MAP image of the tumor region. A threshold was applied to this image to generate a vascular mask (Fig. S14(b)). The Vascular Area Density (VAD)—defined as the ratio of the vascular area to the imaging area—was calculated using this mask. Similarly, the Vessel Complexity Index (VCI), computed as the square of the vascular perimeter divided by the product of the vascular area and 4π, was also derived from the mask. Fig. S14(c) displays the skeleton extracted from the vascular mask, which was used to calculate both the Vessel Skeleton Density (VSD)—defined as the vascular skeleton length divided by the imaging area—and the Vessel Diameter Index (VDI), defined as the vascular area divided by the vascular skeleton length. Figs. S14(d), S14(e), and S14(f) show the PA MAP image, vascular mask, and vascular skeleton for the normal region, respectively.
