# Supplementary material for: A handheld photoacoustic microscopic probe integrating a transparent ultrasound transducer and a fiber scanner
Source: Nat Commun. 2025 Dec 31;17:1409. doi: 10.1038/s41467-025-68148-8 (PMC12881618; doi:10.1038/s41467-025-68148-8)
Supplement: Supplementary file 2 — Description of Additional Supplementary Files [file 41467_2025_68148_MOESM2_ESM.pdf]

Description of Additional Supplementary Files

Supplementary Movie 1: Imaging video of rat's abdominal organ

Supplementary Movie 2: 3D rendered video of rat's stomach vasculature

Supplementary Movie 3: Monitoring video of mouse ear's epinephrine-induced vasoconstriction

Supplementary Movie 4: Monitoring video of mouse ear's lymphatic vessels after Evans blue injection
