# Supplementary material for: A handheld photoacoustic microscopic probe integrating a transparent ultrasound transducer and a fiber scanner
Source: Nat Commun. 2025 Dec 31;17:1409. doi: 10.1038/s41467-025-68148-8 (PMC12881618; doi:10.1038/s41467-025-68148-8)
Supplement: Supplementary file 7 — Reporting Summary [file 41467_2025_68148_MOESM7_ESM.pdf]

nature portfolio

Corresponding author(s): Chulhong Kim

Last updated by author(s): Nov 14, 2025

Reporting Summary

Nature Portfolio wishes to improve the reproducibility of the work that we publish. This form provides structure for consistency and transparency in reporting. For further information on Nature Portfolio policies, see our Editorial Policies and the Editorial Policy Checklist.

Statistics

For all statistical analyses, confirm that the following items are present in the figure legend, table legend, main text, or Methods section.

n/a	Confirmed	
☐	☒	The exact sample size (*n*) for each experimental group/condition, given as a discrete number and unit of measurement
☒	☐	A statement on whether measurements were taken from distinct samples or whether the same sample was measured repeatedly
☒	☐	The statistical test(s) used AND whether they are one- or two-sided<br>*Only common tests should be described solely by name; describe more complex techniques in the Methods section.*
☐	☒	A description of all covariates tested
☒	☐	A description of any assumptions or corrections, such as tests of normality and adjustment for multiple comparisons
☐	☒	A full description of the statistical parameters including central tendency (e.g. means) or other basic estimates (e.g. regression coefficient) AND variation (e.g. standard deviation) or associated estimates of uncertainty (e.g. confidence intervals)
☒	☐	For null hypothesis testing, the test statistic (e.g. *F*, *t*, *r*) with confidence intervals, effect sizes, degrees of freedom and *P* value noted<br>*Give P values as exact values whenever suitable.*
☒	☐	For Bayesian analysis, information on the choice of priors and Markov chain Monte Carlo settings
☒	☐	For hierarchical and complex designs, identification of the appropriate level for tests and full reporting of outcomes
☒	☐	Estimates of effect sizes (e.g. Cohen's *d*, Pearson's *r*), indicating how they were calculated

Our web collection on statistics for biologists contains articles on many of the points above.

Software and code

Policy information about availability of computer code

Data collection	Experimental data were collected using LabVIEW 2017 SP1 (National Instruments)
Data analysis	MATLAB R2023b and R2024a (MathWorks, Inc.) were utilized. 3D PHOVIS application on MATLAB R2023b were utilized.

For manuscripts utilizing custom algorithms or software that are central to the research but not yet described in published literature, software must be made available to editors and reviewers. We strongly encourage code deposition in a community repository (e.g. GitHub). See the Nature Portfolio guidelines for submitting code & software for further information.

Data

Policy information about availability of data

All manuscripts must include a data availability statement. This statement should provide the following information, where applicable:

- Accession codes, unique identifiers, or web links for publicly available datasets
- A description of any restrictions on data availability
- For clinical datasets or third party data, please ensure that the statement adheres to our policy

All data supporting the findings of this study are available within the paper and its Supplementary Information. Additional data are available from the corresponding author upon request and responses to data requests will be provided within one month.

Research involving human participants, their data, or biological material

Policy information about studies with human participants or human data. See also policy information about sex, gender (identity/presentation), and sexual orientation and race, ethnicity and racism.

Reporting on sex and gender	This study did not involve human participants, their data, or biological material
Reporting on race, ethnicity, or other socially relevant groupings	This study did not involve human participants, their data, or biological material
Population characteristics	This study did not involve human participants, their data, or biological material
Recruitment	This study did not involve human participants, their data, or biological material
Ethics oversight	This study did not involve human participants, their data, or biological material

Note that full information on the approval of the study protocol must also be provided in the manuscript.

Field-specific reporting

Please select the one below that is the best fit for your research. If you are not sure, read the appropriate sections before making your selection.

☒ Life sciences ☐ Behavioural & social sciences ☐ Ecological, evolutionary & environmental sciences

For a reference copy of the document with all sections, see nature.com/documents/nr-reporting-summary-flat.pdf

Life sciences study design

All studies must disclose on these points even when the disclosure is negative.

Sample size	The sample size (n=5) was determined in accordance with the 3R principles (Replacement, Reduction, Refinement) of the Institutional Animal Care and Use Committee (IACUC), representing the minimum number of animals required to achieve statistical significance.
Data exclusions	Data were excluded when epinephrine injections were not accurately administered.
Replication	We identified that the technical accuracy of the epinephrine injection was a critical variable. Instances where epinephrine injections were technically inaccurate (e.g., leakage from the injection site or incorrect placement) were not reproducible. All experiments performed with technically accurate injections were successfully replicated.
Randomization	Animals were randomly assigned.
Blinding	Blinding was not applied, as it was not necessary for this experiment.

Reporting for specific materials, systems and methods

We require information from authors about some types of materials, experimental systems and methods used in many studies. Here, indicate whether each material, system or method listed is relevant to your study. If you are not sure if a list item applies to your research, read the appropriate section before selecting a response.

Materials & experimental systems

n/a	Involved in the study	
☒	☐	Antibodies
☒	☐	Eukaryotic cell lines
☒	☐	Palaeontology and archaeology
☐	☒	Animals and other organisms
☒	☐	Clinical data
☒	☐	Dual use research of concern
☒	☐	Plants

Methods

n/a	Involved in the study	
☒	☐	ChIP-seq
☒	☐	Flow cytometry
☒	☐	MRI-based neuroimaging

Animals and other research organisms

Policy information about studies involving animals; ARRIVE guidelines recommended for reporting animal research, and Sex and Gender in Research

Laboratory animals	Experiments were performed using 6-week-old female BALB/c nude mice, 6-week-old female BALB/c nude mic, and 8-week-old female Sprague Dawley rats.
Wild animals	This study did not involve wild animals.
Reporting on sex	All experiments were performed using female animals only; sex was not considered as a variable and no sex-based analyses were conducted.
Field-collected samples	This study did not involve samples collected from the field.
Ethics oversight	All animal experiments were conducted in compliance with the laboratory animal protocol approved by the Institutional Animal Care and Use Committee of Pohang University of Science and Technology (POSTECH-2024-0025).

Note that full information on the approval of the study protocol must also be provided in the manuscript.

Plants

Seed stocks	This study did not use plants.
Novel plant genotypes	This study did not use plants.
Authentication	This study did not use plants.
